# Supplementary material for: Diversity of the Pacific Ocean coral reef microbiome
Source: Nat Commun. 2023 Jun 1;14:3039. doi: 10.1038/s41467-023-38500-x (PMC10235103; doi:10.1038/s41467-023-38500-x)
Supplement: Supplementary file 1 — Supplementary Information [file 41467_2023_38500_MOESM1_ESM.pdf]

Diversity of the Pacific Ocean coral reef microbiome

Supplementary Figures

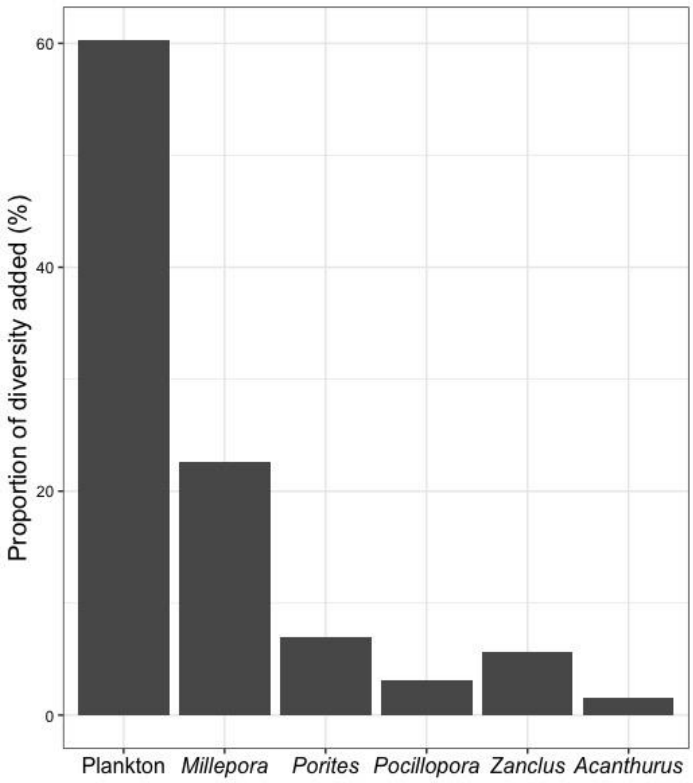

Supplementary Fig. 1. Proportion of microbial diversity added to the accumulation curve (Fig. 1) when adding additional biomes.

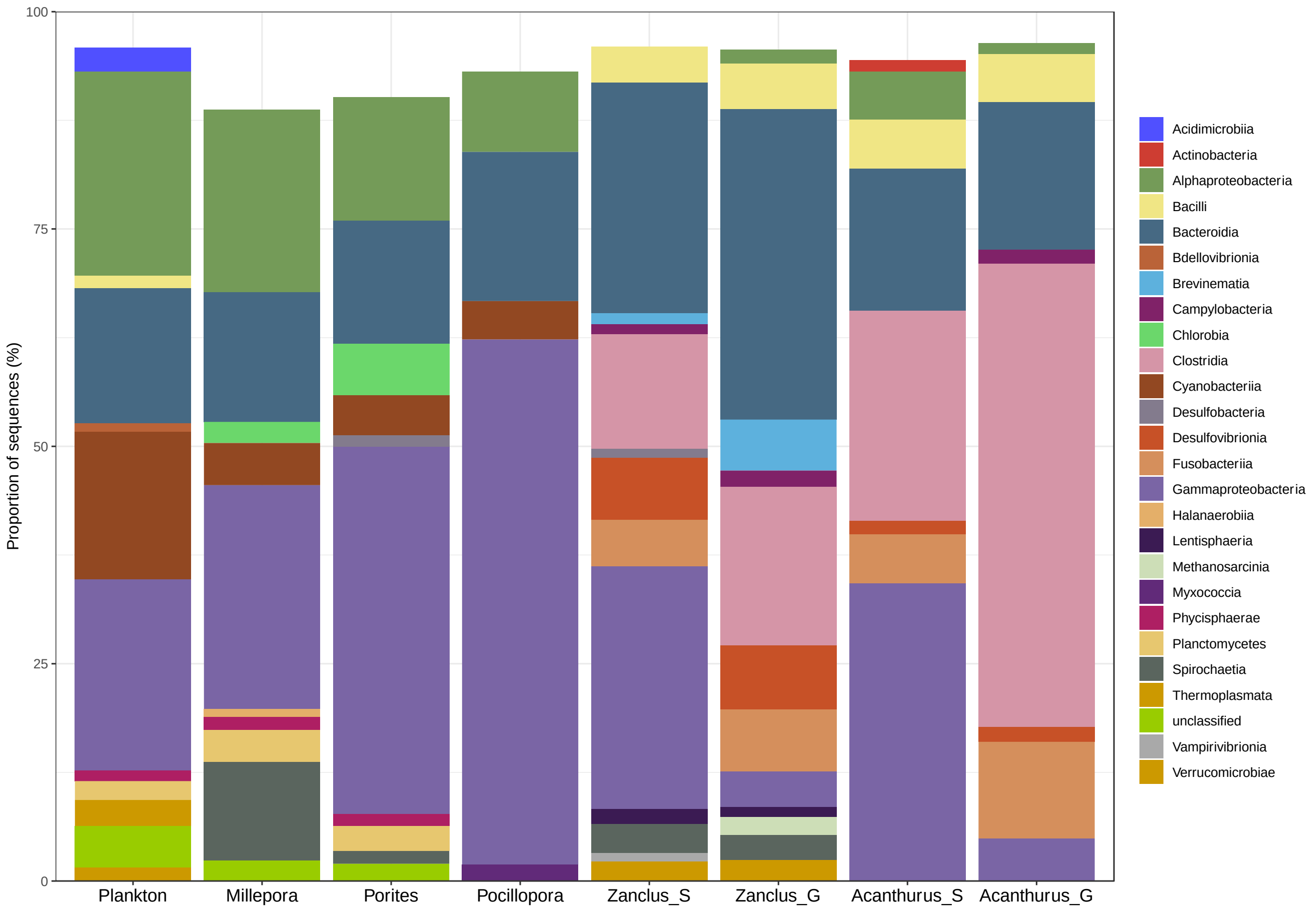

Supplementary Fig. 2. Taxonomic composition at the class level. Classes with abundance >1% in each biome are shown. Plankton includes all size fractions (0.2-3 µm n=336, 3-20 µm n=334, 20 µm n=100, 300 µm n=100). For *Zanclus* and *Acanthurus*, the data is separated in skin (S) and gut (G) samples.

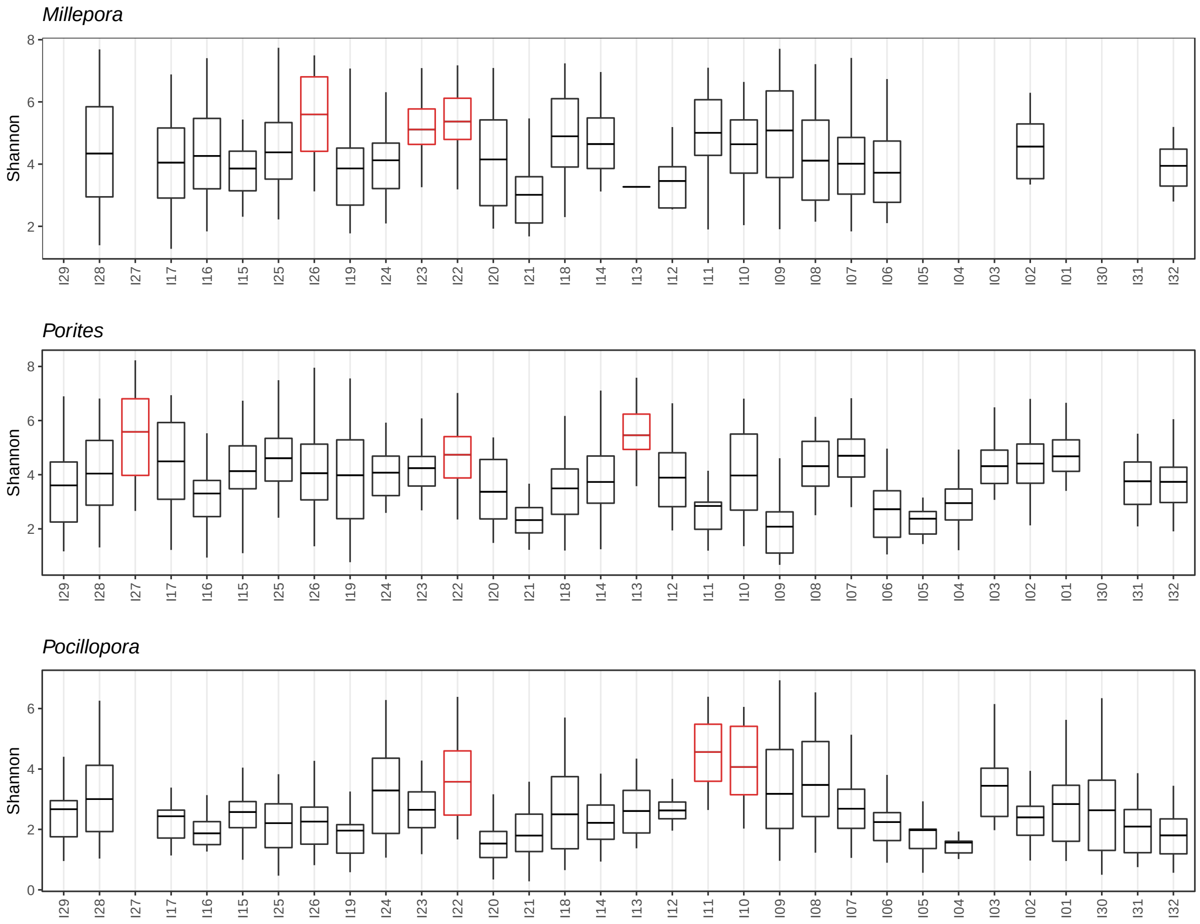

Supplementary Fig. 3: Boxplots showing the value of the Shannon index at all sites for each island for *Millepora* n=619, *Porites* n=945 and *Pocillopora* n=976. Red boxes highlight the 3 most diverse islands for each coral. Islands are ordered from west to east and grouped according to Longhurst provinces. The box plot horizontal bars show the median value, the box indicates the first and third QRs, and the whiskers indicate 1.5*IQR.

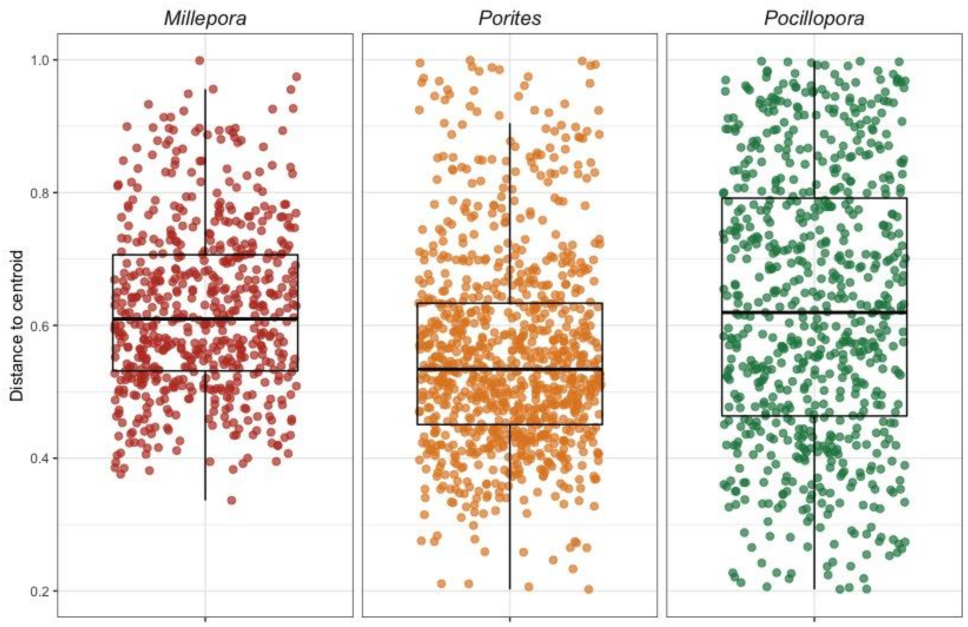

Supplementary Fig. 4. Beta-dispersion (distances of each community to its respective group centroid) of the microbial communities of *Millepora* n=619, *Porites* n=945 and *Pocillopora* n=976. The box plot horizontal bars show the median value, the box indicates the first and third QRs, and the whiskers indicate 1.5*IQR.

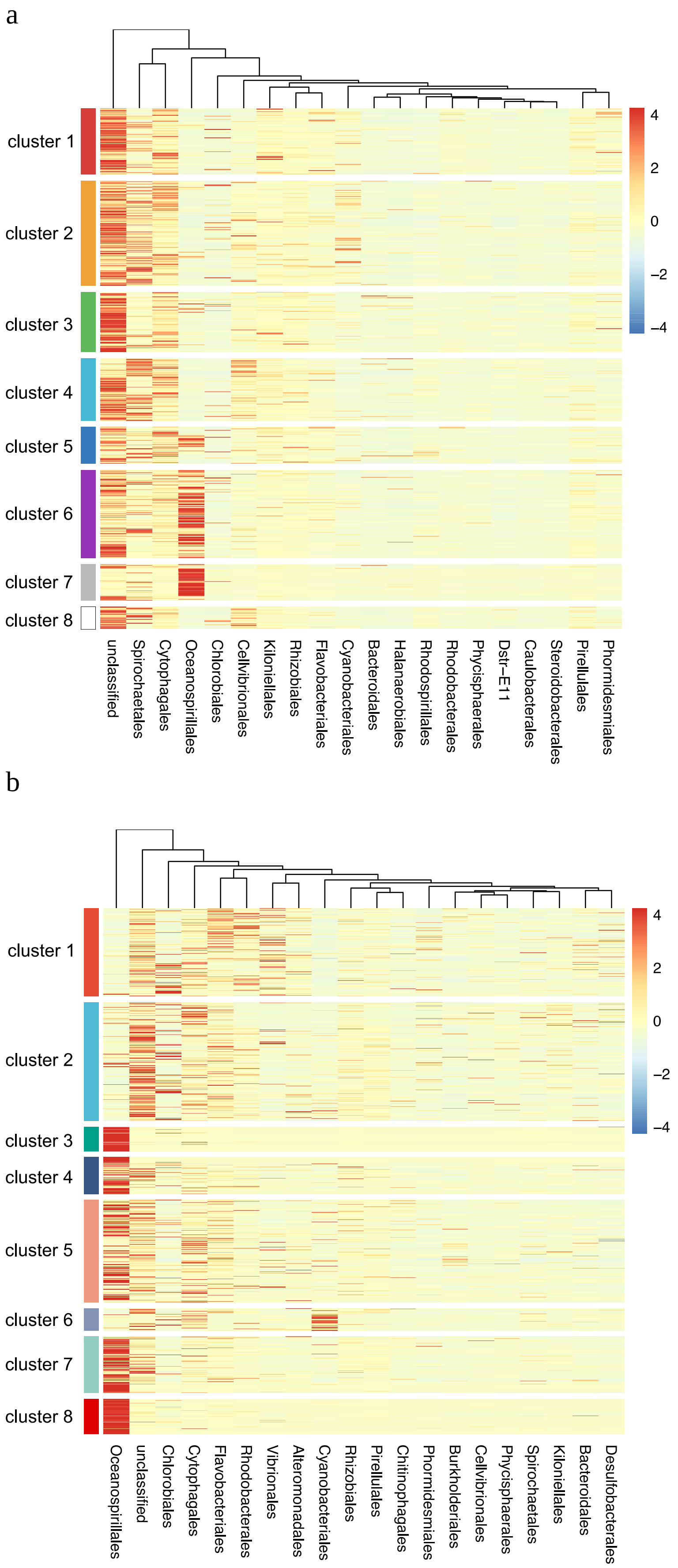
a
cluster 1
cluster 2
cluster 3
cluster 4
cluster 5
cluster 6
cluster 7
cluster 8
4
2
0
−2
−4
unclassified
Spirochaetales
Cytophagales
Oceanospirillales
Chlorobiales
Cellvibrionales
Kiloniellales
Rhizobiales
Flavobacteriales
Cyanobacteriales
Bacteroidales
Halanaerobiales
Rhodospirillales
Rhodobacterales
Phycisphaerales
Dstr−E11
Caulobacterales
Steroidobacterales
Pirellulales
Phormidesmiales
b
cluster 1
cluster 2
cluster 3
cluster 4
cluster 5
cluster 6
cluster 7
cluster 8
4
2
0
−2
−4
Oceanospirillales
unclassified
Chlorobiales
Cytophagales
Flavobacteriales
Rhodobacterales
Vibrionales
Alteromonadales
Cyanobacteriales
Rhizobiales
Pirellulales
Chitinophagales
Phormidesmiales
Burkholderiales
Cellvibrionales
Phycisphaerales
Spirochaetales
Kiloniellales
Bacteroidales
Desulfobacterales

c

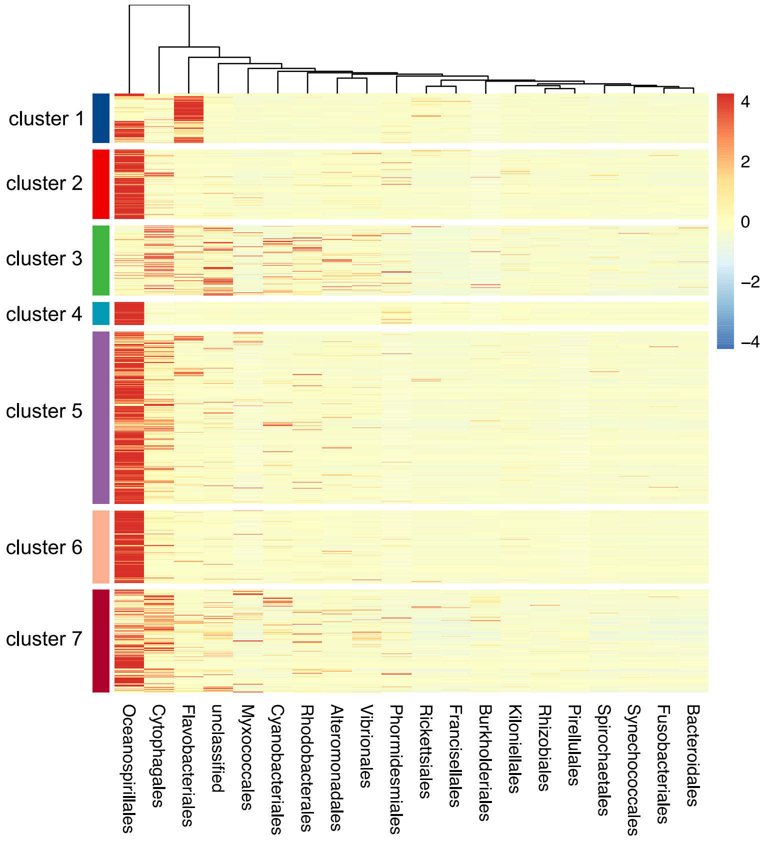

Supplementary Fig. 5. Heatmap showing the clustering of coral samples (rows) against the 20 most abundant bacterial orders (columns) for each coral morphotype: **a** *Millepora*, **b** *Porites*, and **c** *Pocillopora*. The community cluster colour code corresponds to the ones in Fig. 5.

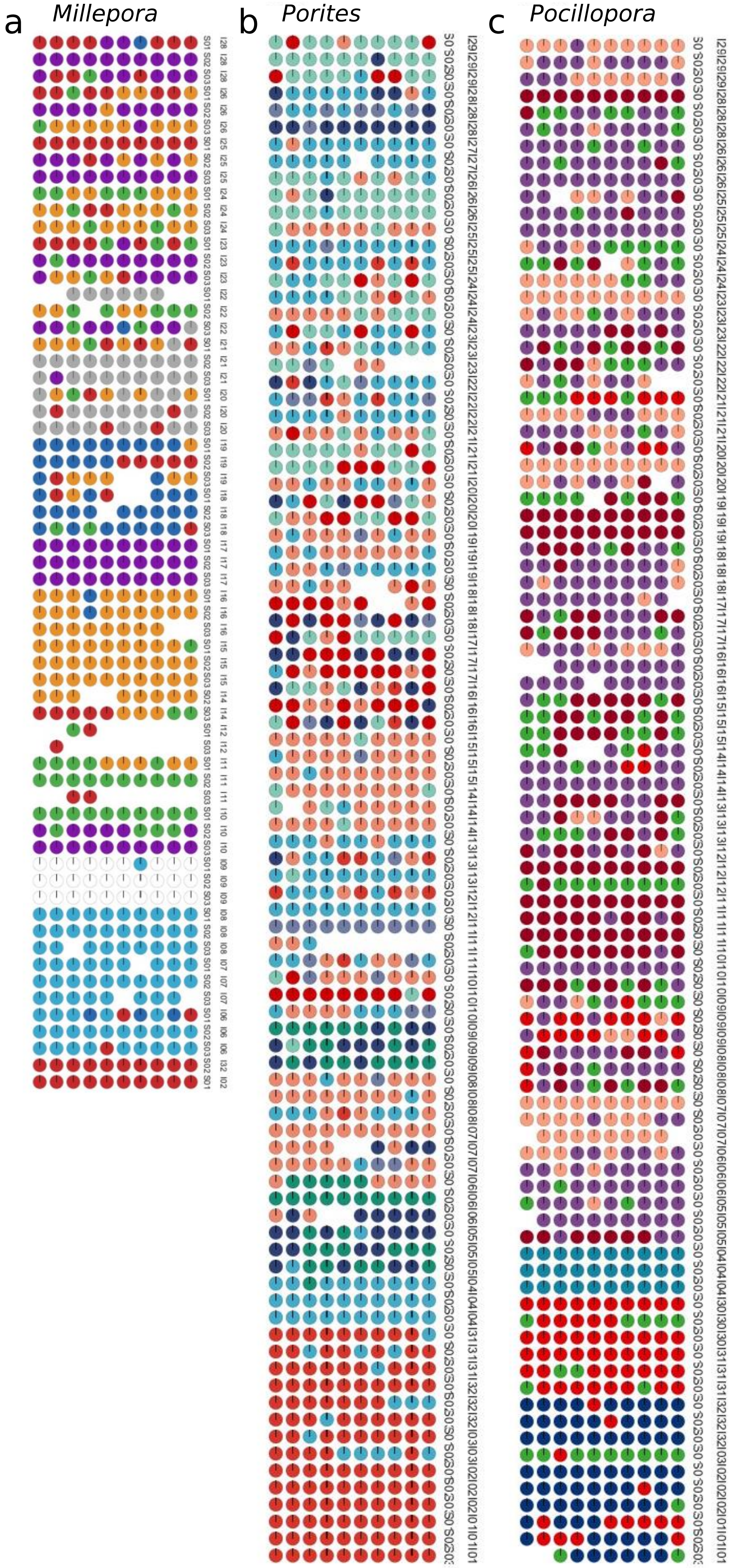
a
Millepora
b
Porites
c
Pocillopora

Supplementary Fig. 6. Microbial community composition across the islands (I01 to I32) sampled in the Pacific Ocean for *Millepora* (a), *Porites* (b) and *Pocillopora* (c). The pie charts represent the different community clusters identified by hierarchical clustering (see method). Similar colours within a figure panel represent similar microbial communities. Data for 3 sites (S01, S02 and S03) at each island are represented. An average of 10 different colonies were sampled at each site. The relative proportion of the community clusters at each site was used to identify groups of islands with similar microbial communities (Fig. 4).

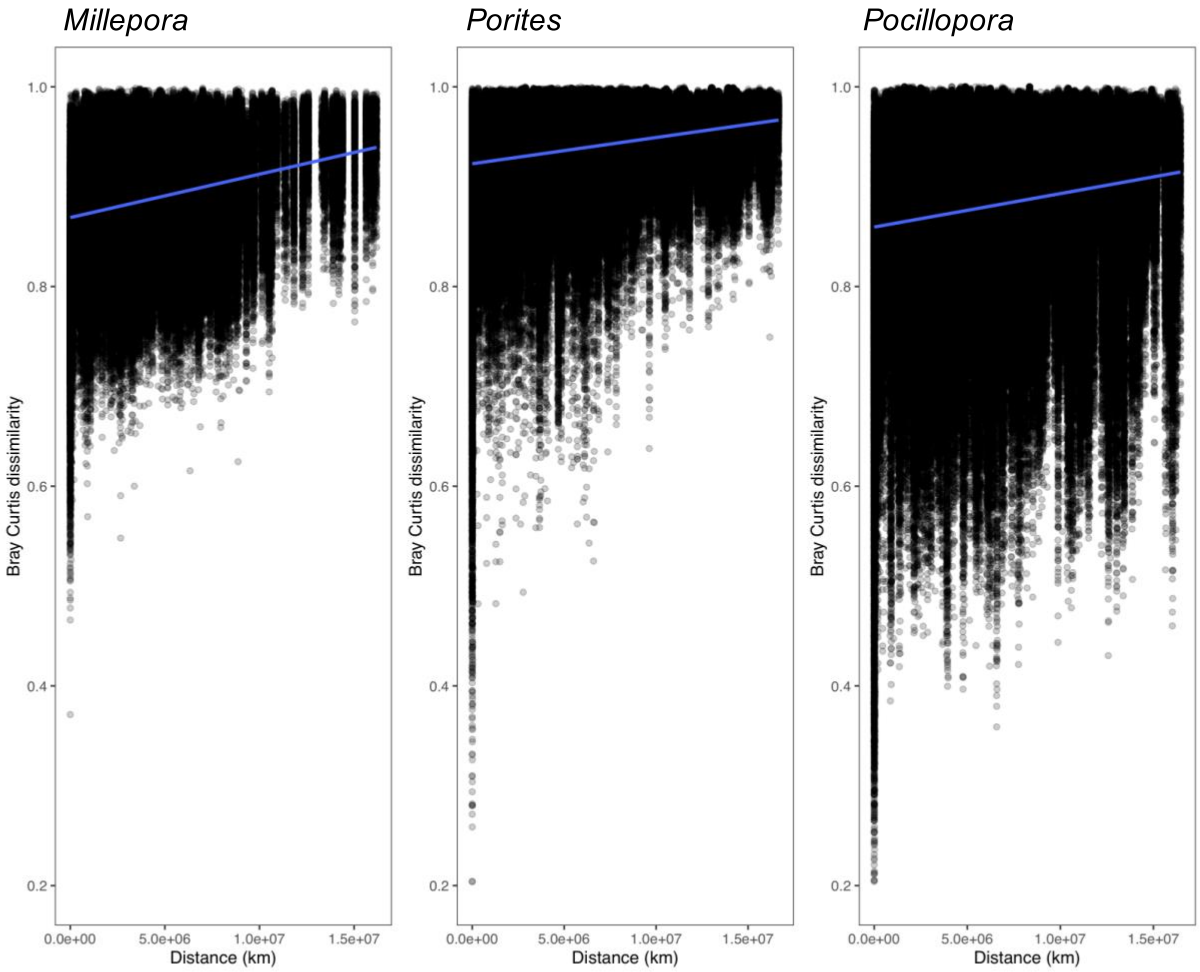

Supplementary Fig. 7. Relationship between the geographical distance separating two samples and community composition for *Millepora*, *Porites* and *Pocillopora*. Linear regression is indicated by a blue line.

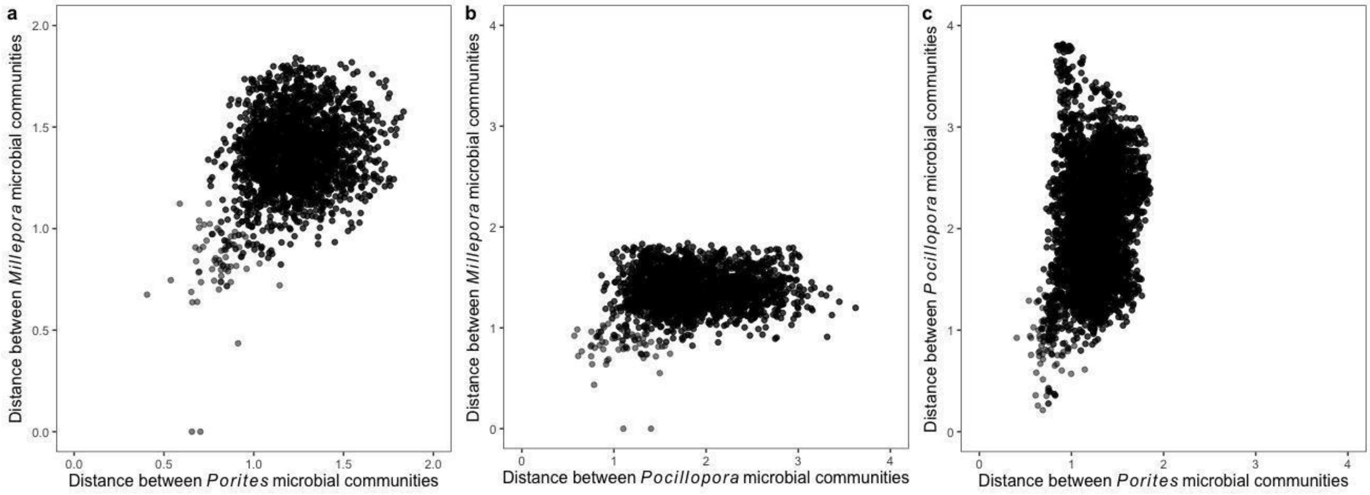

Supplementary Fig. 8. Pairwise dissimilarity of microbial communities between sampling sites. Comparison of the pairwise dissimilarity of microbial communities between sites for the corals **a** *Porites* versus dissimilarity among *Millepora*, **b** *Pocillopora* versus *Millepora*, and **c** *Porites* versus *Pocillopora*.

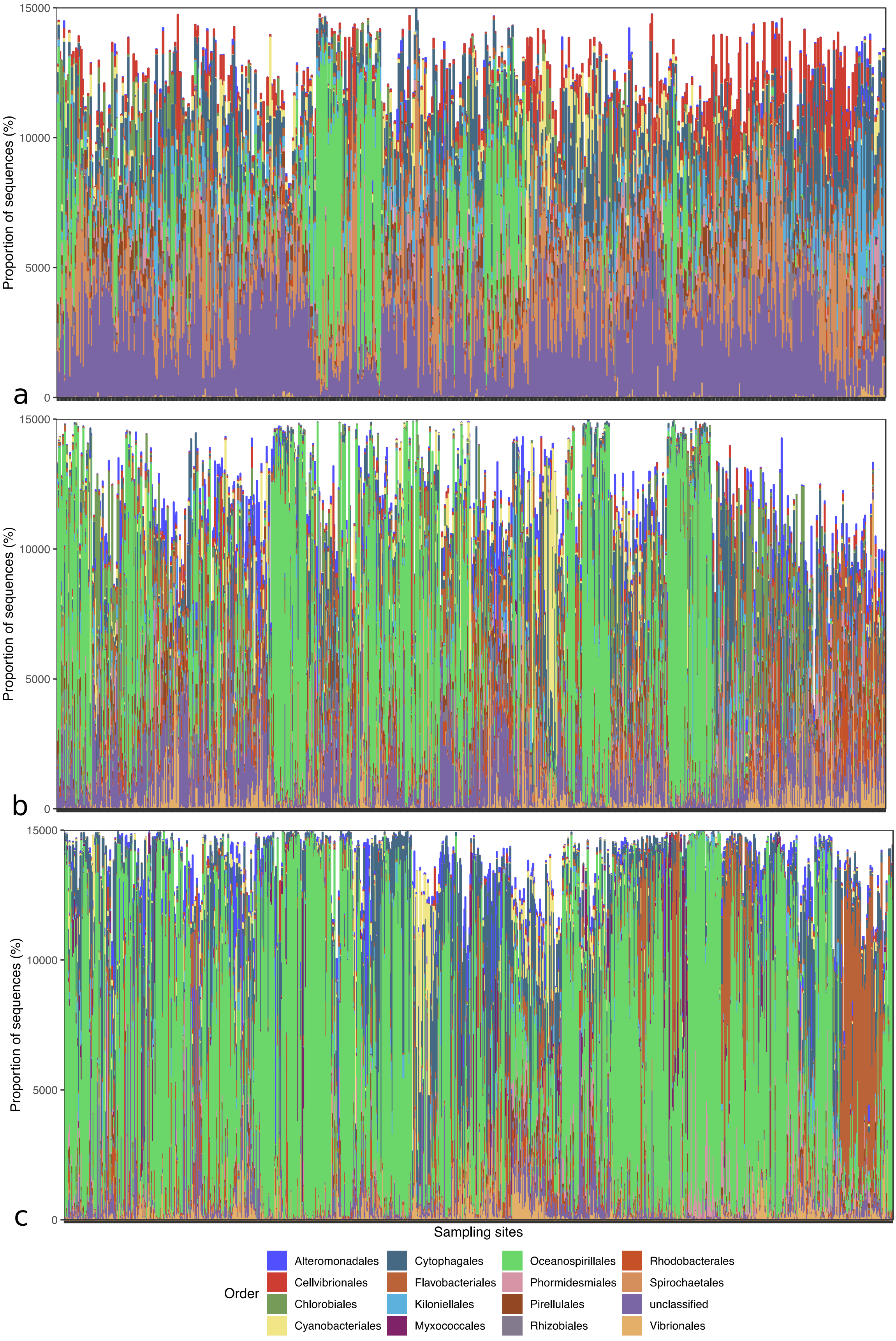

Supplementary Fig. 9. Community composition for the 10 most abundant bacterial orders in *Millepora* (a), *Porites* (b) and *Pocillopora* (c) at all sampling sites. Sites are ordered by Longhurst provinces and sorted from the west (left) to the east (right) Pacific Ocean.

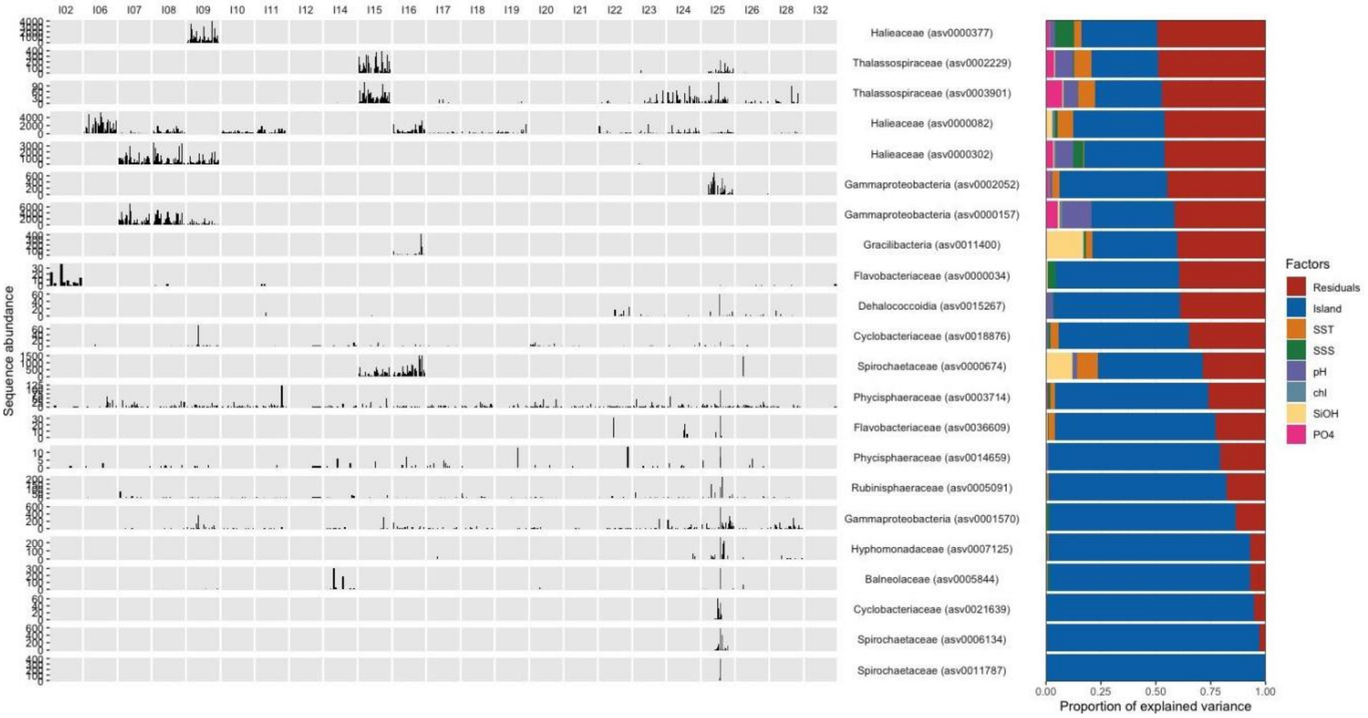

Sequence abundance
Halieaceae (asv0000377)
Thalassospiraceae (asv0002229)
Thalassospiraceae (asv0003901)
Halieaceae (asv0000082)
Halieaceae (asv0000302)
Gammaproteobacteria (asv0002052)
Gammaproteobacteria (asv0000157)
Gracilibacteria (asv0011400)
Flavobacteriaceae (asv0000034)
Dehalococcoidia (asv0015267)
Cyclobacteriaceae (asv0018876)
Spirochaetaceae (asv0000674)
Phycisphaeraceae (asv0003714)
Flavobacteriaceae (asv0036609)
Phycisphaeraceae (asv0014659)
Rubinisphaeraceae (asv0005091)
Gammaproteobacteria (asv0001570)
Hyphomonadaceae (asv0007125)
Balneolaceae (asv0005844)
Cyclobacteriaceae (asv0021639)
Spirochaetaceae (asv0006134)
Spirochaetaceae (asv0011787)
Proportion of explained variance
Factors
Residuals
Island
SST
SSS
pH
chl
SiOH
PO4

a

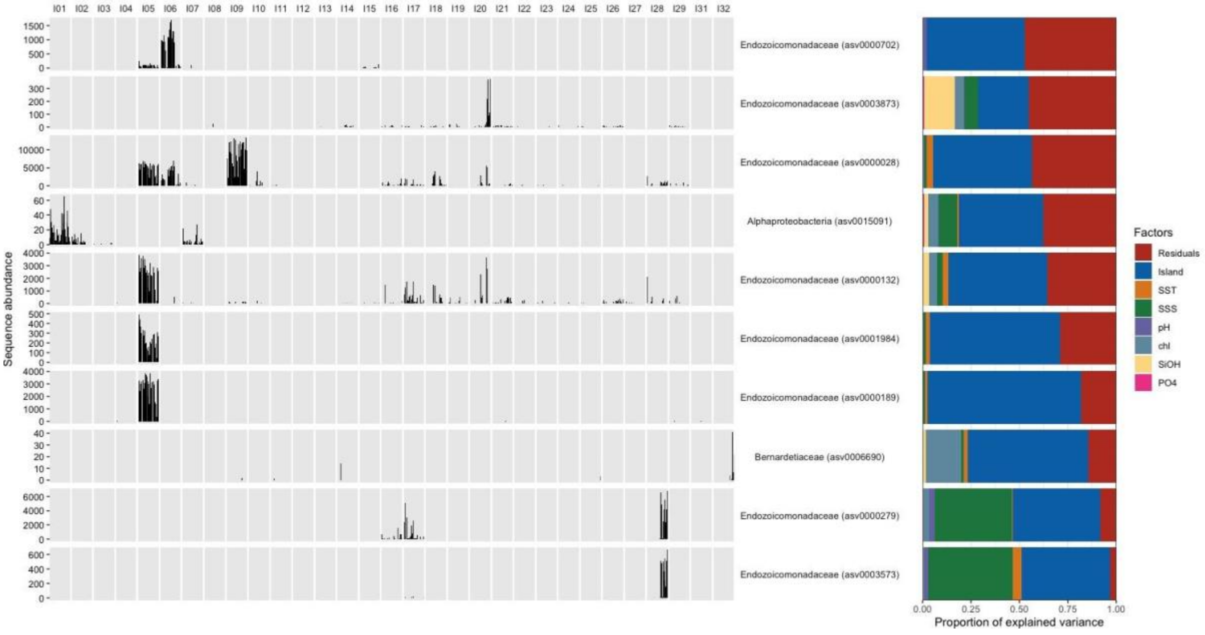

Sequence abundance
Endozoicomonadaceae (asv0000702)
Endozoicomonadaceae (asv0003873)
Endozoicomonadaceae (asv0000028)
Alphaproteobacteria (asv0015091)
Endozoicomonadaceae (asv0000132)
Endozoicomonadaceae (asv0001984)
Endozoicomonadaceae (asv0000189)
Bernardetiaceae (asv0006690)
Endozoicomonadaceae (asv0000279)
Endozoicomonadaceae (asv0003573)
Proportion of explained variance
Factors
Residuals
Island
SST
SSS
pH
chl
SiOH
PO4

b

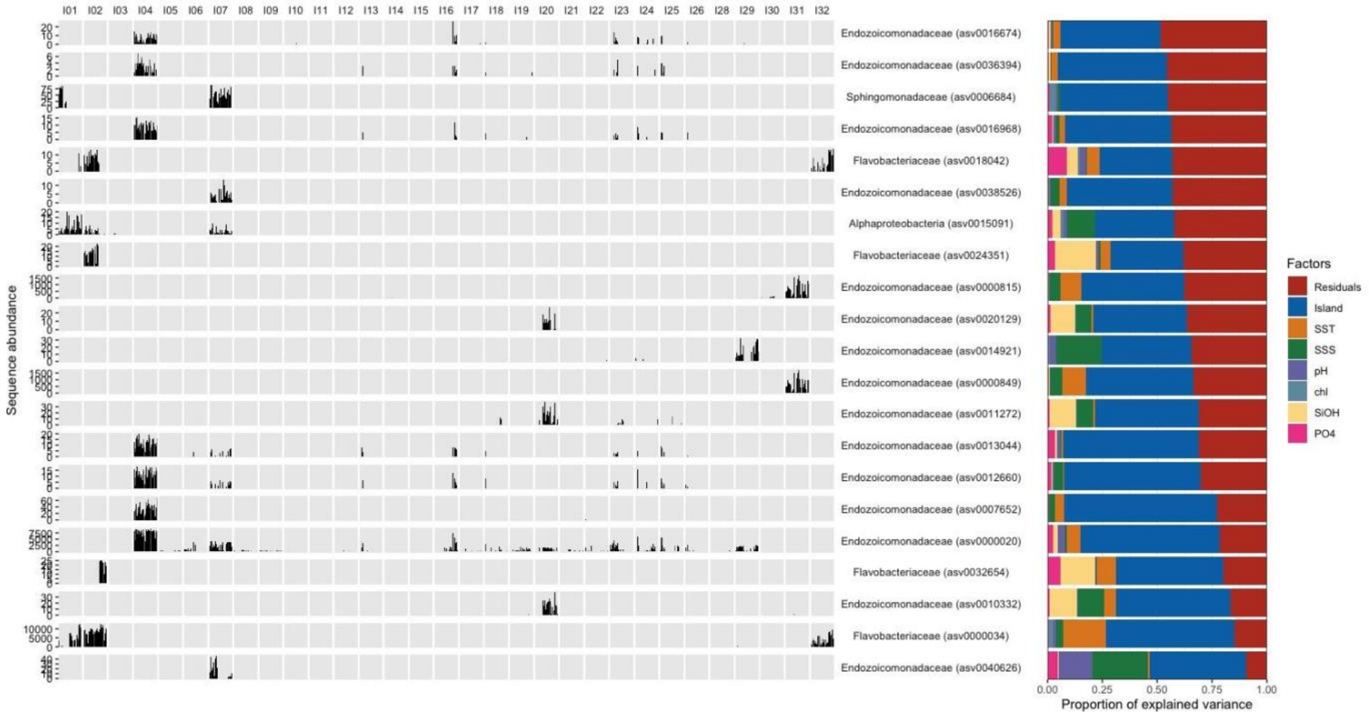

c

Supplementary Fig. 10. Relative sequence abundance across coral colonies for the ASVs that had the most variance explained by island and environmental factors (residuals <50%) (left panel), and representation of the proportion of the variance explained by the different factors (right panel) for *Millepora* (a), *Porites* (b) and *Pocillopora* (c). The taxonomy of the ASVs is given at the family level or higher level when unclassified at the family level.

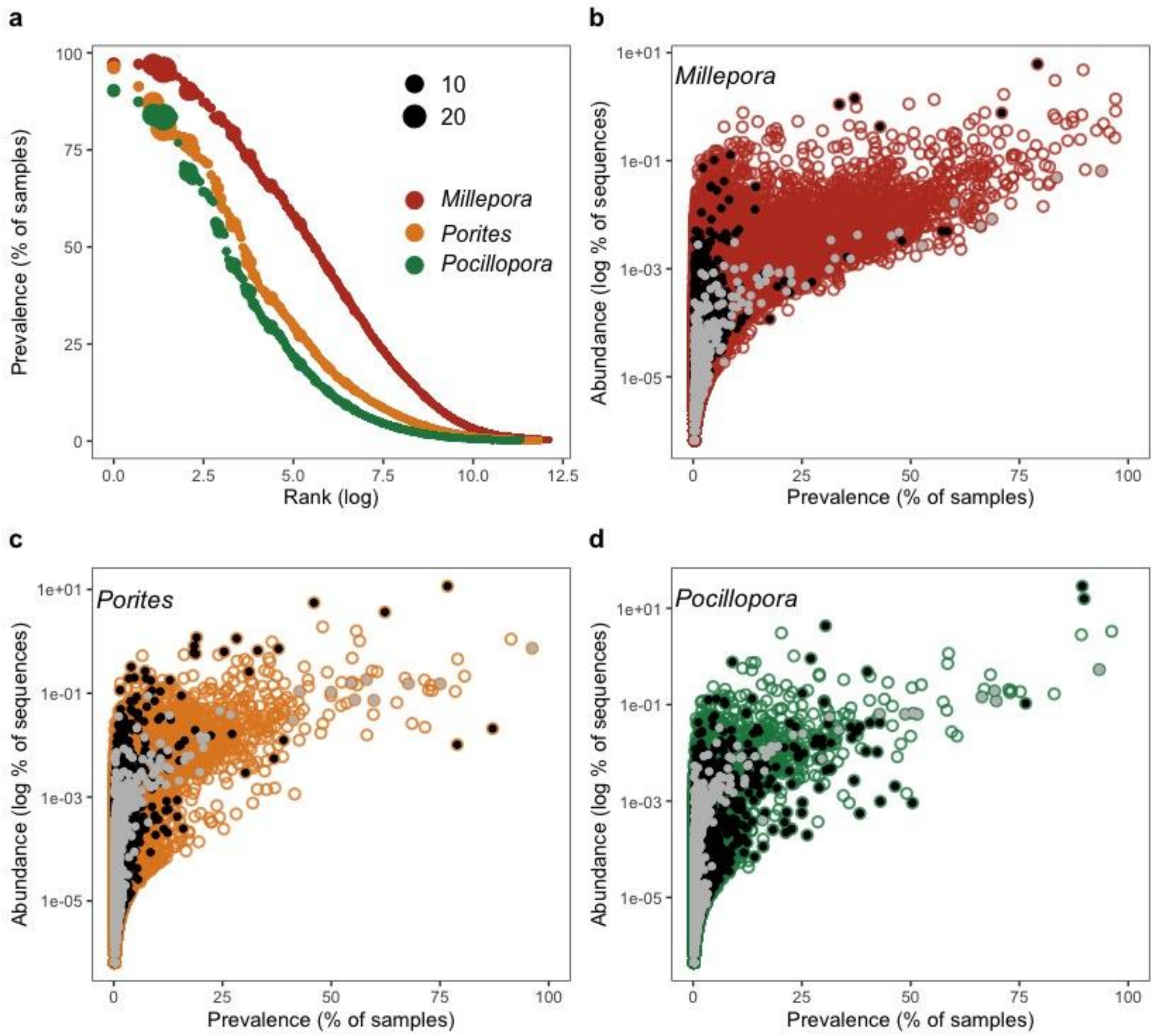

Supplementary Fig. 11. Prevalence (percentage of samples found in) and abundance (average relative abundance within samples) of prokaryotic ASVs in all 3 coral genera. **a** Rank prevalence curve for the 3 coral genera. The size of the dots represents the relative abundance of the ASV within a coral genus. **b** Prevalence versus abundance of ASVs in *Millepora*, **c** in *Porites* and **d** in *Pocillopora*. Black dots represent ASV annotated as Endozoicomonadaceae, grey show Vibrionaceae and all other annotations are white. The colours of the circles correspond to the 3 coral genera.

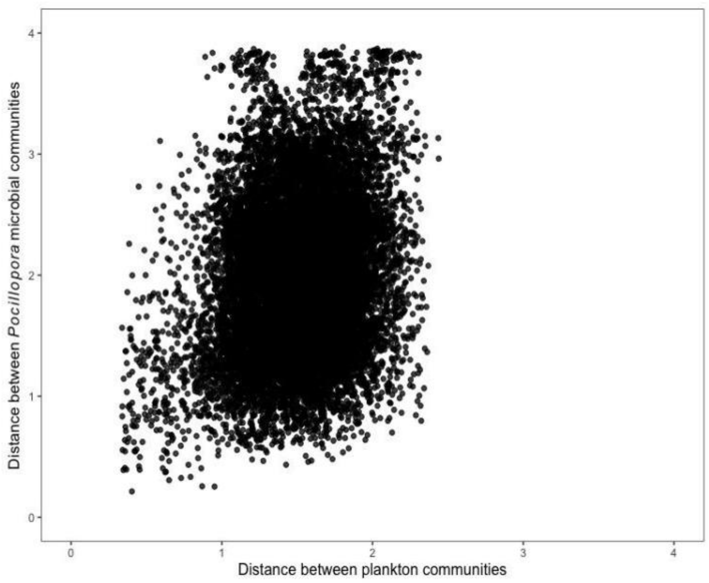

Supplementary Fig. 12. Pairwise dissimilarity between microbial communities compared between Pocillopora microbial communities and free-living planktonic communities (size 0.2 – 3µm) sampled close to the *Pocillopora* colonies (colony water).

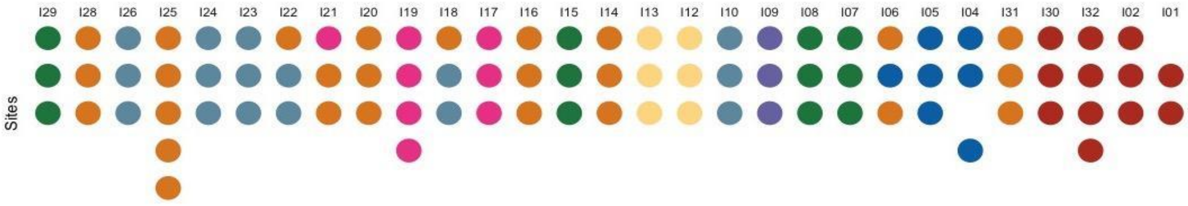

Supplementary Fig. 13. Microbial community composition across the Pacific Ocean for free-living planktonic communities (size 0.2 – 3µm) sampled close to the *Pocillopora* colonies (colony water). The pie charts represent the proportion of the different community clusters identified by hierarchical clustering (see method).

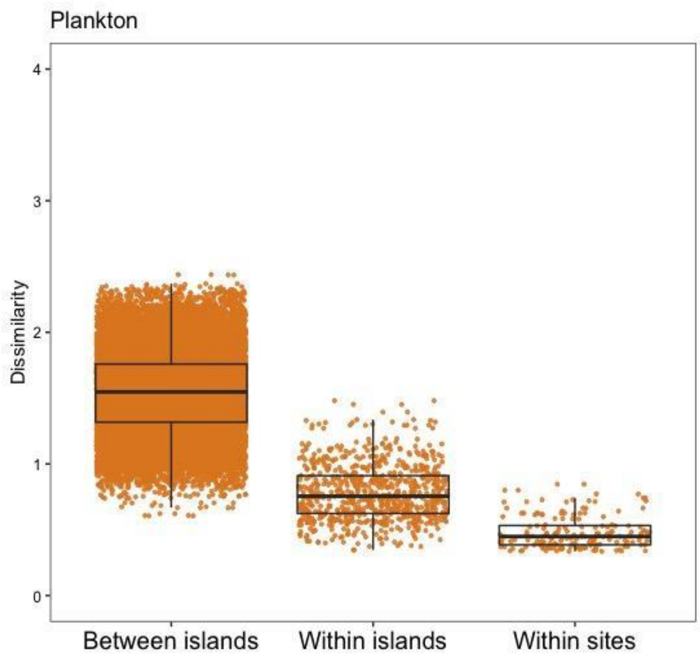

Supplementary Fig. 14. Pairwise dissimilarity between microbial communities compared between islands, within islands and within sites at each island for free-living planktonic communities (size 0.2 – 3µm) sampled close to the *Pocillopora* colonies (colony water), n=30,276 comparisons. The box plot horizontal bars show the median value, the box indicates the first and third QRs, and the whiskers indicate 1.5*IQR. The dissimilarity is based on Euclidean distance computed from centred log ratio (clr) transformed data.
