# Supplementary material for: Diversity of the Pacific Ocean coral reef microbiome
Source: Nat Commun. 2023 Jun 1;14:3039. doi: 10.1038/s41467-023-38500-x (PMC10235103; doi:10.1038/s41467-023-38500-x)
Supplement: Supplementary file 3 — Description of Additional Supplementary Files [file 41467_2023_38500_MOESM3_ESM.pdf]

Description of Additional Supplementary Files

File Name: Supplementary Data 1.

Description: Prevalence of ASVs across the 3 coral genera
