# Supplementary material for: Diversity of the Pacific Ocean coral reef microbiome
Source: Nat Commun. 2023 Jun 1;14:3039. doi: 10.1038/s41467-023-38500-x (PMC10235103; doi:10.1038/s41467-023-38500-x)
Supplement: Supplementary file 5 — Reporting Summary [file 41467_2023_38500_MOESM5_ESM.pdf]

nature portfolio

Corresponding author(s): Pierre Galand
Last updated by author(s): Feb 16, 2023

Reporting Summary

Nature Portfolio wishes to improve the reproducibility of the work that we publish. This form provides structure for consistency and transparency in reporting. For further information on Nature Portfolio policies, see our Editorial Policies and the Editorial Policy Checklist.

Statistics

For all statistical analyses, confirm that the following items are present in the figure legend, table legend, main text, or Methods section.

n/a	Confirmed	
☐	☒	The exact sample size (*n*) for each experimental group/condition, given as a discrete number and unit of measurement
☐	☒	A statement on whether measurements were taken from distinct samples or whether the same sample was measured repeatedly
☐	☒	The statistical test(s) used AND whether they are one- or two-sided<br>*Only common tests should be described solely by name; describe more complex techniques in the Methods section.*
☐	☒	A description of all covariates tested
☐	☒	A description of any assumptions or corrections, such as tests of normality and adjustment for multiple comparisons
☐	☒	A full description of the statistical parameters including central tendency (e.g. means) or other basic estimates (e.g. regression coefficient) AND variation (e.g. standard deviation) or associated estimates of uncertainty (e.g. confidence intervals)
☐	☒	For null hypothesis testing, the test statistic (e.g. *F*, *t*, *r*) with confidence intervals, effect sizes, degrees of freedom and *P* value noted<br>*Give P values as exact values whenever suitable.*
☒	☐	For Bayesian analysis, information on the choice of priors and Markov chain Monte Carlo settings
☒	☐	For hierarchical and complex designs, identification of the appropriate level for tests and full reporting of outcomes
☒	☐	Estimates of effect sizes (e.g. Cohen's *d*, Pearson's *r*), indicating how they were calculated

Our web collection on statistics for biologists contains articles on many of the points above.

Software and code

Policy information about availability of computer code

Data collection	NA
Data analysis	codes for sequence analysis in DADA2 v1.14 are published in zenodo: https://zenodo.org/record/4073035, other analysis were conducted in R packages: BiodiversityR v2.12.3, vegan v2.5.7, rtk v0.2.6.1, easycoda v0.34.3, NbClust v3.0, geosphere v1.5.10, variancePartition v1.20.0

For manuscripts utilizing custom algorithms or software that are central to the research but not yet described in published literature, software must be made available to editors and reviewers. We strongly encourage code deposition in a community repository (e.g. GitHub). See the Nature Portfolio guidelines for submitting code & software for further information.

Data

Policy information about availability of data

All manuscripts must include a data availability statement. This statement should provide the following information, where applicable:

- Accession codes, unique identifiers, or web links for publicly available datasets
- A description of any restrictions on data availability
- For clinical datasets or third party data, please ensure that the statement adheres to our policy

Sequence data are available from zenodo: https://zenodo.org/record/4073035 and environmental data: https://zenodo.org/record/6299409. All sequencing files were submitted to the European Nucleotide Archive (ENA) at the EMBL European Bioinformatics Institute (EMBL-EBI) under the Tara Pacific Umbrella BioProject PRJEB47249. All other data supporting the findings of this study are provided in the Supplementary Information/Source Data file.

Human research participants

Policy information about studies involving human research participants and Sex and Gender in Research.

Reporting on sex and gender	NA
Population characteristics	NA
Recruitment	NA
Ethics oversight	NA

Note that full information on the approval of the study protocol must also be provided in the manuscript.

Field-specific reporting

Please select the one below that is the best fit for your research. If you are not sure, read the appropriate sections before making your selection.

☐ Life sciences ☐ Behavioural & social sciences ☒ Ecological, evolutionary & environmental sciences

For a reference copy of the document with all sections, see nature.com/documents/nr-reporting-summary-flat.pdf

Ecological, evolutionary & environmental sciences study design

All studies must disclose on these points even when the disclosure is negative.

Study description	Analysis of the diversity of the microbiome associated to coral reef across the Pacific Ocean
Research sample	Coral colonies from 3 genera (Millepora, Porites, Pocillopora). They represent 3 different lineages of corals. They were chosen because all are present across the entire Pacific Ocean.
Sampling strategy	10 replicate colonies of each coral genus sampled at 3 different sites at each of the 32 targeted islands. This very large number of replicates, never achieved earlier, was chosen to give a precise picture of the diversity.
Data collection	Data collected by scuba diving as precisely described in Lombard, F. et al. Open science resources from the Tara Pacific expedition across coral reef and surface ocean ecosystems. bioRxiv (2022)
Timing and spatial scale	Samples collected aross the Pacific Ocean during the Tara Pacific expedition from 2016 to 2018.
Data exclusions	Data from non-target coral genus were excluded
Reproducibility	NA
Randomization	Not relevant because this a field based study
Blinding	Not relevant because this a field based study

Did the study involve field work? ☒ Yes ☐ No

Field work, collection and transport

Field conditions	All environmental parameters are given in Zenodo: https://zenodo.org/record/6299409
Location	99 different reefs from 32 island systems across the entire Pacific Ocean
Access & import/export	Sampling and export follow international rules for obtaining sampling permits. Authorization for sampling was provided under the following sampling permits (UNCLOS): for PANAMA under the reference 'SE/AP-18-16' delivered by the Direccion de Areas Protegidas y Vida Silvestre - LIC. Samuel Valdez Diaz Director - Ministerio de Ambiente – Republica de Panama on the 13/06/2016; Sampling permit for PANAMA under the reference '2016-0701-2019-2' delivered by the Smithsonian Tropical Research Institute Instituto Smithsonian de Investigaciones Tropicales - STRI Animal Care and Use Committee (ACUC) on the 28/06/2016; Sampling permit for PANAMA under the reference '2016-0701-2019-2-A1' delivered by the Smithsonian Tropical Research Institute Instituto Smithsonian de Investigaciones Tropicales - STRI Animal Care and Use Committee (ACUC) on the 21/06/2018; Sampling permit for COOK under the reference '11-16' delivered by the Foundation for National Research – Cook Island Research Committee – Office of the Prime

	Minister – Elizabeth Wright-Koteka (Chairperson) on the 12/09/2016; Sampling permit for NIUE under the reference '34/16' delivered by the Government of Niue – Office for External Affairs on the 17/11/2016; Sampling permit for SAMOA under the reference 'Memorandum of Agreement' delivered by the THE GOVERNMENT OF THE INDEPENDENT STATE OF SAMOA acting by and through the Ministry of Natural Resources and Environment on the 29/11/2016; Sampling permit for WALLIS AND FUTUNA under the reference 'Arrêté n°2016-527' delivered by the Le Préfet, Administrateur supérieur des îles Wallis et Futuna on the 24/11/2016; Sampling permit for TUVALU under the reference 'MFAT : 449/16' delivered by the Government of Tuvalu – Ministry of Foreign Affairs on the 19/12/2016; Sampling permit for KIRIBATI under the reference '015/16' delivered by the Environment and Conservation Division – Republic of Kiribati on the 24/11/2016; Sampling permit for MICRONESIA under the reference 'Letter' delivered by the Deputy Assistant Secretary – Marine Resources Unit – Department of Resources and Development – Federated States of Micronesia on the 05/04/2017; Sampling permit for GUAM under the reference 'U2021-023' delivered by the Marine Scientific Research Coordinator Office of Ocean and Polar Affairs – United States Department of State Bureau of Oceans and International Environmental and Scientific Affairs on the 27/10/2021; Sampling permit for AMERICAN SAMOA under the reference 'U2021-022' delivered by the Marine Scientific Research Coordinator Office of Ocean and Polar Affairs – United States Department of State Bureau of Oceans and International Environmental and Scientific Affairs on the 27/10/2021; Sampling permit for JAPAN (Tokyo Prefecture; Ogasawara Island) under the reference '28-50' delivered by the Prefecture of Tokyo on the 01/23/2017; Sampling permit for JAPAN (Okinawa Prefecture; Sesoko Island) under the reference '28-74' delivered by the Prefecture of Okinawa on the 04/14/2017; Sampling permit for JAPAN (Japanese EEZ) under the reference 'N/A' delivered by the Ministry of Agriculture, Forestry and Fisheries on the 01/10/2017; Sampling permit for FIJI under the reference '456/2017' delivered by the Ministry of Foreign Affairs – Republic of Fiji on the 11/06/2017; Sampling permit for AUSTRALIA under the reference 'G17/39873.1' delivered by the Great Barrier Reef Marine Park Authority and Department of Foreign Affairs and Trade on the 30/08/2017; Sampling permit for NEW-CALEDONIA (SOUTH PROVINCIA) under the reference 'Arrêté n°2720-2017/ARR/DENV modifiant l'arrêté 1515-2017/ARR/DENV du 04 août 2017' delivered by the Président de l'Assemblée de la Province Sud de la Nouvelle-Calédonie on the 06/09/2017; Sampling permit for NEW-CALEDONIA (CHESTERFIELD) under the reference 'Arrêté n°2017-2069/GNC' delivered by the Haut-Commissariat de la République en Nouvelle-Calédonie – Gouvernement de Nouvelle-Calédonie – République Française on the 29/08/2017; Sampling permit for SOLOMON ISLANDS under the reference 'Form 01' delivered by the Solomon Islands Maritime Safety Administration on the 20/09/2017; Sampling permit for PAPUA NEW-GUINEA under the reference '907/2017 (diplomatic clearance n°0232)' delivered by the Department of Foreign Affairs and Trade of the Independent State of Papua New Guinea on the 27/10/2017; Sampling permit for PALAU under the reference 'RE-18-04' delivered by the Ministry of Natural Resources, Environment and Tourism – Republic of Palau on the 21/12/2017; Sampling permit for TAIWAN (Pingtung county) under the reference '10707821600' delivered by the Pingtung Agri-Fish; National Taiwan Ocean University on the 06/04/2018; Sampling permit for TAIWAN (Taitung county) under the reference '1070033041' delivered by the Taitung Agri-Fish; National Taiwan Ocean University on the 12/02/2018; Sampling permit for USA (HAWAII) under the reference 'U2018-010' delivered by the United States Department of State Bureau of Oceans and International Environmental and Scientific Affairs on the 06/06/2018; Sampling permit for MEXICO under the reference 'PPF/DGOPA-291/17' delivered by the Secrataria de Agricultura, Ganaderia, Desarrollo rural, pesca y alimentacion – Comision Nacional de Acuacultura y Pesca – Direccion General de Ordenamiento Pesquero y Acuicola – Estados Unidos Mexicanos on the 28/08/2018; Sampling permit for CLIPPERTON under the reference 'HC/1195/CAB' delivered by the Haut-Commissariat de la République Polynésie Française on the 13/06/2018.
Disturbance	Disturbance was minimized by minimizing all contacts with the corals.

Reporting for specific materials, systems and methods

We require information from authors about some types of materials, experimental systems and methods used in many studies. Here, indicate whether each material, system or method listed is relevant to your study. If you are not sure if a list item applies to your research, read the appropriate section before selecting a response.

Materials & experimental systems

n/a	Involved in the study
☒	☐ Antibodies
☒	☐ Eukaryotic cell lines
☒	☐ Palaeontology and archaeology
☒	☐ Animals and other organisms
☒	☐ Clinical data
☒	☐ Dual use research of concern

Methods

n/a	Involved in the study
☒	☐ ChIP-seq
☒	☐ Flow cytometry
☒	☐ MRI-based neuroimaging
